# Pharmaceutical Cost-Saving Strategies and their Association with Medication Adherence in a Medicare Supplement Population

**DOI:** 10.1007/s11606-015-3196-7

**Published:** 2015-02-10

**Authors:** Shirley Musich, Yan Cheng, Shaohung S. Wang, Cynthia E. Hommer, Kevin Hawkins, Charlotte S. Yeh

**Affiliations:** 1 Advanced Analytics, Optum, 315 E. Eisenhower Parkway, Suite 305, Ann Arbor, MI 48108 USA; 2 MCG – Formerly Milliman Care Guidelines, Seattle, WA USA; 3 AARP Services, Inc., Washington, DC USA

**Keywords:** cost-saving strategies, free drug samples, medication nonadherence, Medicare, Medigap

## Abstract

**BACKGROUND:**

On average, Medicare Supplement insureds take about seven unique prescription medications each year, resulting in substantial out-of-pocket drug copayments, in addition to Medicare Supplement and Part D premiums. To help alleviate this financial burden, many individuals resort to cost-saving strategies that are not trackable by Part D insurance plans, likely resulting in an underestimation of medication adherence rates.

**OBJECTIVE:**

We aimed to estimate utilization rates of cost-saving strategies, measure member characteristics associated with these strategies and estimate if these strategies are associated with medication adherence.

**DESIGN:**

This was a cross-sectional analysis of a 2012–2013 survey of AARP® Medicare Supplement plan insureds with Part D pharmaceutical coverage.

**PARTICIPANTS:**

The study included 5,784 community-dwelling survey respondents ≥ 65 years of age, living in ten states and with self-reported use of prescription medications.

**MAIN MEASURES:**

Self-reported use of cost-saving strategies included: obtaining free samples from physicians, splitting pills so medications lasted longer, purchasing medications from other countries and/or over the internet, or purchasing medications through the Veterans Administration. Propensity weighted multivariate regressions were utilized to determine characteristics associated with the use of such strategies and the association with medication adherence as measured from Medicare Part D claims.

**KEY RESULTS:**

Among those taking medications, 39.6 % used cost-saving strategies. Those using these strategies were significantly (*p* < 0.05) more likely to be male, non-minority, have more comorbid conditions, have more disabilities and use more medications. Few variables were significantly related to pharmaceutical nonadherence, but those who were nonadherent were significantly more likely to use more medications, split pills, obtain free samples from their physicians and be male.

**CONCLUSION:**

Cost-saving strategies are used extensively as a means to augment Medicare Part D coverage. These strategies are associated with measured medication nonadherence and likely result in underreporting of medication adherence rates. Pharmacy management programs should consider these additional medication sources in assisting plan members to problem solve cost-related medication management issues.

## INTRODUCTION

Prescription drugs represent one of the highest out-of-pocket (OOP) healthcare costs for American consumers, accounting for about $47 billion dollars in 2012.^1^ About 90 % of Medicare Supplement insureds take at least one prescription medication,^2^ resulting in substantial OOP drug copayments. Medicare Supplement plans are often referred to as “Medigap” plans because they help to defray the OOP expenses not covered by the fee-for-service Medicare program; Part D insurance provides prescription drug coverage. Among users, Medicare Supplement insureds take about seven unique prescription medications each year, with medication needs greatest for the older age groups and those with multiple chronic conditions.^2^ As a result, 10 % of insureds average over $1,300 in annual OOP spending on prescription medications.^3^

With the implementation of Medicare Part D in 2006, significant improvements in pharmaceutical coverage for the elderly occurred. However, despite improvements in percentages of those with coverage,^4^ documented increases in medication adherence^5^^–^ 7 and significant decreases in OOP spending,^4^^,^ 5 ^,^ 7 ^–^ 10 11 to 26 % of Medicare insureds continue to self-report that they skip doses, split pills or do not fill prescriptions due to cost.^11^^–^ 15 The prevalence of such cost-related nonadherence (CRN) is even higher among minorities, those with lower incomes and those in poorer health.^10^^,^ 13 ^,^ 16 Additional strategies used by older adults to lower OOP spending include other options, such as requesting free samples from physicians, splitting pills to make medications last longer, purchasing medications from other countries and over the internet, or leveraging other sources of insurance coverage (e.g., Medicaid or Department of Veterans Affairs [VA] insurance).^17^^–^ 22 These alternative medication sources are generally not trackable through administrative pharmaceutical claims, which impairs the ability to monitor pharmaceutical adherence by Part D insureds.

Rates of medication nonadherence are highly sensitive to the cost burden associated with OOP spending—as OOP spending increases with higher numbers of medications, medication nonadherence increases.^22^^–^ 28 Other factors, such as demographics (e.g., gender, race), socioeconomics (e.g., income, education), health literacy, depression or other lifestyle behaviors (e.g., physical activity, smoking), are less consistently associated with medication adherence.^9^^,^ 10 ^,^ 16 ^,^ 27 ^–^ 32 Regardless of reasons why seniors do not comply with recommended protocols, medication nonadherence has consistently been associated with negative health outcomes, including increased use of emergency rooms,^13^ hospitalizations and total healthcare costs.^23^^,^ 24 ^,^ 33

Most of the literature examining prescription drug purchasing patterns has focused on general Medicare populations.^17^^–^ 21 We found no studies investigating whether disparities regarding utilization of cost-saving strategies exist among older adults with Medicare Supplement plans (i.e., Medigap). While most (about 90 %) of those with fee-for-service Medicare coverage have some type of supplemental insurance coverage, only about 28 % (about 10.2 million adults) have purchased Medigap coverage.^34^ Thus, those with Medigap coverage represent a significant portion of the Medicare population. We hypothesize that patient demographics, health status and benefit levels likely differ by Medigap source and plan type, and therefore, may impact disparities in the utilization of cost-saving strategies compared to the general Medicare populations. Furthermore, to date, multivariate models estimating significant predictors of medication adherence have not included untrackable cost-savings strategies, such as obtaining free samples from physicians or splitting pills.

Considering the importance of OOP spending to medication adherence, the primary objective of this study was to estimate utilization rates of cost-saving strategies among a Medicare Supplement study population. The secondary objectives included determining member characteristics associated with the use of these strategies and estimating the association of these strategies with medication adherence as measured via part D claims.

## METHODS

### Sample Selection

In 2013, approximately 3.5 million Medicare insureds were covered by an AARP® Medicare Supplement plan insured by UnitedHealthcare Insurance Company (for New York residents, UnitedHealthcare Insurance Company of New York). These plans are offered in all 50 states, Washington D.C. and various U.S. territories. A randomly selected sample of 31,000 of these insureds in ten states (Arizona, California, Colorado, Florida, Missouri, North Carolina, Ohio, New Jersey, New York and Texas) was surveyed in 2012 and 2013. The sampling strategy included an eligibility criterion for care management programs (i.e., oversampling those with more intensive health needs).

Those surveyed were 65 years or older with a minimum of 3 months of AARP Supplement plan eligibility. To be included in this study, survey respondents were also required to have purchased AARP Medicare Part D prescription drug coverage. Those who indicated that they were not currently taking prescription medications (8 %) or who didn’t answer the survey question on cost savings (2 %) were excluded. The final study sample included 5,784 survey respondents. This study sample was used to examine prevalence of utilization of cost-saving strategies and characteristics associated with the use of these strategies.

To determine medication adherence, survey respondents were linked to Evidence-Based Medicine (Symmetry EBM Connect® Version 7.6) software. This software program was developed to calculate medication adherence based on pharmaceutical claims. Medication adherence calculations were limited to those patients with a minimum of one year of continuous medical enrollment, six months of pharmaceutical drug coverage and at least two prescriptions for a given medication with an associated diagnosis (e.g., diabetes). Ten common primary chronic conditions aligned with those queried on the survey were included in this analysis (see Table 1 for a list of conditions). A subgroup of 4,222 survey respondents was utilized for the medication adherence analysis. To be considered “adherent,” individuals must have had at least 70 % of the days supplied for the medications within a given therapeutic drug class. Therapeutic drug classes are a way of classifying prescription drugs according to their functions in treating similar medical conditions. We then counted the number of medications for which each individual was nonadherent across all categories of prescription medications.

**Table 1:** Chronic Conditions Utilized for Medication Adherence

Chronic conditions	
Asthma	
Atrial Fibrillation	
Chronic Obstructive Pulmonary Disease	
Congestive Heart Failure	
Coronary Artery Disease	
Depression	
Diabetes Mellitus	
Hyperlipidemia	
Hypertension	
Osteoporosis Management	

### CAHPS Survey

The Consumer Assessment of Healthcare Providers and Systems (CAHPS) is a survey funded and overseen by the U.S. Agency for Healthcare Research and Quality (AHRQ). The survey is designed to query patients and consumers to report on and evaluate their experiences and satisfaction with Medicare delivery systems. The survey is in the public domain and has become the national standard for measuring and reporting on patient experiences.

This self-reported survey measures the member’s demographics, socioeconomic factors, health status and perception of experiences and satisfaction with the different components of healthcare services. The CAHPS survey was adapted for distribution to our population to identify their health needs and inform potential interventions. In this study, the following three questions relevant to our primary and secondary objectives were used:

1. Please mark…each type of health insurance you have in addition to Medicare and your Medicare Supplement Insurance Plan: Veteran’s Benefits also known as VA benefits; Employer, Union or Retiree health coverage (insurance); Medicare prescription drug plan or other insurance.
2. “Do you now need or take medicine prescribed by your doctor?” Yes/No
3. “In the last 6 months, did you ever do any of the following to save money on your medications? (Check all that apply).”
  Use mail order
  Use generic medications
  Purchase medications over the internet
  Purchase medication from other countries
  Purchase medications from retail outlets
  Obtain free samples from doctors
  Split pills so medications last longer
  Did something else
  I did not have difficulty paying for medications

Cost-saving strategies were defined as using any of the following: purchasing medications over the internet or from other countries; obtaining free samples from doctors; splitting pills to make medications last longer and using other sources of insurance coverage (e.g., VA insurance). Using mail order, generic medications, purchasing from retail outlets and doing something else were not included as cost-saving strategies. When repayment is not due, retail pharmacy chains often do not consistently report sales of prescription drugs (e.g., $4 generic drugs) to pharmacy-benefit managers (PBMs). Since our study design included an impact of these strategies on medication adherence from administrative pharmacy databases, we would not have been able to interpret the adherence modeling results had this variable been included. Mail order and generic medications are generally tracked in administrative pharmacy databases. Doing something else included too few individuals to be analyzed as a separate category.

### Covariates

Covariates were included to adjust for factors that may have influenced the outcomes. These covariates included survey measures of demographics, socioeconomic factors, health status and other characteristics taken from health plan eligibility and claims files. Demographic questions included age, gender, race and state of residence. Socioeconomic questions measured living arrangements and education levels (from the survey) and income levels (geocoded from zip codes to high, upper medium, lower medium or low income levels). Health status included body mass index (BMI) categorized to underweight (BMI < 18.5), normal (BMI 18.5–24.9), overweight (BMI 25–29) or obese (BMI 30+), smoking status, general physical health and self-reported treatment of common chronic conditions. Modified Katz Activities of Daily Living (ADLs; difficulties with bathing, dressing, eating, transferring in and out of a chair, walking and using the toilet) were assessed as a measure of disability. ADL disabilities requiring assistance were categorized as: none, one, two or three or more. Other sources of drug insurance coverage included employer insurance or VA insurance. The survey also included a question on whether the person needed help to complete the survey and an assessment of confidence in filling out medical forms, both of which could be considered proxies for health literacy and/or health condition (e.g., disability). The demographic, socioeconomic and health status covariates considered are listed in Table 1.

### Propensity Weighting for Survey Non-Response Bias

Propensity weighting was used to adjust for the potential selection bias often associated with survey response, to enhance the generalizability of these findings. The propensity weighting utilized available information about the demographic, socioeconomic and health status variables described above that could potentially influence survey response. This information was used to estimate the underlying probability of survey response for each individual; that estimated probability was then used to create a weighting variable applied to the data from those who chose not to respond, to make them better resemble all eligible insureds. The utility of such propensity weighting models to adjust for external validity threats is described elsewhere.^35^^,^ 36

### Statistical Models

Survey respondents were categorized into two possible groups, based on their response to the cost-saving strategies question: 1) those who utilized cost-saving strategies or 2) those who reported that they did not need or use such strategies. Propensity weighted multivariate logistic regression modeling was used to determine significant characteristics associated with utilization of cost-saving strategies, adjusted for covariates listed in Table 2.

**Table 2:** Characteristics of the Cost-Saving Strategies Study Sample (Unadjusted)

	Overall *N* = 5,784	Used Cost-Saving Strategies *N* = 2,293	Did Not Use Cost-Saving Strategies *N* = 3,491	p value
	%	%	%	
Gender				
Male	39.8	44.1	37.0	< 0.0001
Race				
White	84.6	85.2	84.3	0.33
Age group (years)				
65–69	16.0	15.5	16.4	0.38
70–74	24.3	24.9	23.9	0.35
75–79	20.4	21.7	19.6	0.06
80–84	18.3	18.1	18.4	0.80
85 or more	21.0	19.8	21.8	0.07
Income (geocoded from zip code)				
High	56.4	57.0	56.0	0.47
Upper middle	22.5	22.2	22.7	0.67
Lower middle	14.9	14.3	15.3	0.27
Lower	6.2	6.5	5.9	0.38
Education				
High school or less or missing	34.5	30.3	37.3	< 0.0001
Some college/2 year college	25.9	27.5	24.9	0.03
4 year college or more	28.3	31.0	26.6	0.0003
Living arrangement				
Personal home or apartment	83.3	83.7	83.0	0.49
Body Mass Index (BMI)				
Under weight (BMI < 18.5)	2.3	1.9	2.6	0.06
Normal weight or missing (BMI 18.5–24.9)	30.2	28.8	31.2	0.05
Over weight (BMI 25–29)	31.5	30.9	31.9	0.41
Obese (BMI 30 +)	23.9	26.5	22.2	0.0002
Help completing survey				
Yes	8.7	9.6	8.1	0.04
Treated medical conditions				
Heart problems	23.2	28.6	19.6	< 0.0001
Stroke	2.2	2.9	1.8	0.007
Lung problems	14.7	21.1	10.5	< 0.0001
Digestive problems	8.3	10.9	6.6	< 0.0001
Musculoskeletal problems	32.0	40.2	26.6	< 0.0001
Diabetes	20.1	25.0	17.0	< 0.0001
Depression	7.2	7.8	6.8	0.14
Cancer	10.7	14.3	8.3	<0.0001
Ever engaged in care management programs				
Yes	32.0	35.8	29.5	0.004
Insurance status (self-reported)				
Employer’s insurance	3.2	3.4	3.0	0.44
Medicare Part D insurance	74.2	75.1	73.6	0.20
Department of Veterans Affairs insurance	2.6	6.7	0.0	< 0.0001
General physical health				
Excellent/very good	29.4	24.0	33.0	< 0.0001
Good	43.2	43.2	43.1	0.94
Poor/fair	27.4	32.8	23.9	< 0.0001
Smoking				
Yes	4.1	3.9	4.3	0.44
Activities of daily living (ADLs) requiring assistance				
No ADLs	49.9	43.4	54.1	< 0.0001
One ADL	14.9	15.4	14.6	0.35
Two ADLs	12.6	15.0	11.0	< 0.0001
Three or more ADLs	17.9	21.6	15.4	< 0.0001
Have confidence completing medical forms				
Yes	79.1	77.2	80.4	0.004
Have difficulty paying for drugs				
No	38.4	26.6	46.2	< 0.0001
Number of therapeutic drug classes				
0–3	12.8	12.8	22.1	< 0.0001
4–5	22.6	19.2	24.8	< 0.0001
6–7	21.9	23.8	20.7	0.005
8–9	16.9	18.5	15.9	0.008
10 or more	20.2	25.7	16.5	< 0.0001

State of residence (AZ, CA, CO, FL, MO, NJ, NY, NC, OH and TX) was included in the demographics, but not listed in the table for brevity

For the medication adherence analysis, propensity weighted negative binomial regression modeling was used to determine significant characteristics associated with medication nonadherence (i.e., count of nonadherent medications) adjusted for covariates listed in Table 2. Negative binomial models are commonly used to analyze outcomes, such as event counts, that have positive integer values with skewed distributions.^37^

## RESULTS

The overall response rate for the survey was 31 % (*N* = 9,708). After the inclusion criteria (including self-reported use of prescription medications and Medicare Part D eligibility) were applied, 66 % of survey respondents qualified for the study (*N* = 5,784). Of those, 4,222 (73 %) had required administrative prescription drug claims to be included in the medication adherence modeling. The unadjusted characteristics of the study sample are shown in Table 2. Overall, survey respondents were mostly female (60.2 %), 75 years or older (59.7 %), highly educated (54.2 % some college or more), with high income (56.4 %) and of white race (84.6 %). The most common chronic conditions were high blood pressure (48.1 %) and musculoskeletal problems (32.0 %). Most respondents reported good health (39.8 %). Overall, characteristics of those in the adherence analysis were similar (data not shown but available upon request).

Among eligible respondents, 39.6 % indicated they used at least one of five queried strategies to save money on medications. Most reported obtaining free samples from doctors (29.8 %) or splitting pills so medications would last longer (12.6 %). Over 38 % reported that they did not have difficulty paying for medications (Table 3).

**Table 3:** Strategies to Save Money on Medications (*N* = 5,784)

Strategies	%
Cost-Saving Strategies	
Obtain free samples from doctors	29.8
“Split pills” so medications last longer	12.6
Purchase medications from other countries	3.5
Purchase medications over the internet	2.7
Department of Veterans Affairs insurance coverage	2.6
Any of the above cost-saving strategies	39.6
Other Saving Strategies	
Use mail order	37.5
Use generic medications	82.2
Purchase medications from retail outlets	24.1
Did something else	2.5
Don’t have difficulty paying for medications	38.4

### Characteristics Associated with Use of Cost-Saving Strategies

The results of the propensity weighted multivariate logistic regression model used to predict characteristics associated with use of cost-saving strategies are shown in Table 4. Those using cost-saving strategies were more likely to be male, highly educated and white race. Those with more chronic conditions, including lung problems, musculoskeletal problems, depression, digestive problems, diabetes and heart problems, were more likely to use cost-saving strategies. Similarly, those using more prescription medications (four or more therapeutic classes of prescription medications) and those with more activities of daily living (ADL) disabilities used the strategies. Those who had no difficulty paying for medications, were underweight, minorities and had upper middle incomes were less likely to use cost-saving strategies.

**Table 4:** Multivariate Logistic Regression Analysis for Use of Cost-Saving Strategies (*N* = 5,784)

Variables	Odds Ratio	p value
No difficulty paying	0.45	< 0.0001
Underweight	0.64	< 0.0001
Minority	0.69	< 0.0001
Upper medium income (geocoded)	0.89	0.004
3+ ADLs	1.13	0.02
Age 70–74 years	1.13	0.01
Ever engaged in care management	1.16	0.001
Heart problems	1.17	< 0.0001
Age 75–79 years	1.17	0.002
Live in personal home	1.19	0.005
4–5 Therapeutic drug classes	1.22	< 0.0001
2 ADLs	1.22	< 0.0001
Employer insurance coverage	1.25	0.02
Depression	1.28	< 0.0001
Diabetes	1.29	< 0.0001
Digestive problems	1.29	< 0.0001
Some college	1.41	< 0.0001
Male	1.42	< 0.0001
Musculoskeletal problems	1.43	< 0.0001
8–9 Therapeutic drug classes	1.51	< 0.0001
College 4+ years	1.58	< 0.0001
6–7 Therapeutic drug classes	1.62	< 0.0001
Lung problems	1.63	< 0.0001
10+ Therapeutic drug classes	1.64	< 0.0001

*ADLs* Activities of Daily Living

Propensity weighted and adjusted for demographic, socioeconomic and health status variables listed in Table 2

### Characteristics Associated with Medication Nonadherence

The results of the propensity weighted negative binomial regression model used to predict characteristics associated with medication nonadherence as measured with Part D claims are shown in Table 5. Few variables were significantly associated to medication nonadherence, but those who were nonadherent were significantly more likely to use more medications, split pills, obtain free samples from their physicians and to be male. Those more likely to adhere with their medication protocols were more likely to be in very good to excellent health and to be younger (i.e., 65–74 years).

**Table 5:** Multivariate Binomial Regression Analysis for Medication Nonadherence (*N* = 4,222)

Variable	Odds Ratio	p value
Age 65–69 years	0.77	0.05
General health very good to excellent	0.80	0.05
Age 70–74 years	0.83	0.10*
Male	1.18	0.03
Obtain free samples from doctors	1.18	0.04
Ever engaged in care management	1.19	0.06*
6–7 Therapeutic drug classes	1.27	0.07*
“Split pills” so medications last longer	1.45	0.001
8–9 Therapeutic drug classes	1.46	0.006
10+ Therapeutic drug classes	1.65	<0.0001

*Borderline significant *p* < 0.10; Propensity weighted and adjusted for demographic, socioeconomic and health status variables listed in Table 2

## DISCUSSION

In our study, Medicare Supplement insureds spent on average $820 OOP on prescription medications, not including Medicare Supplement and Part D premiums. Among those taking medications, 39.6 % utilized cost-saving strategies to reduce OOP spending on prescription medications. The most commonly used cost-saving strategy was obtaining free samples from physicians (29.8 %) followed by splitting pills to make medications last longer (12.6 %). Those who used cost-saving strategies were more likely to be male, white, more highly educated, have more chronic conditions, more disabilities and to take more medications. Consequently, those less likely to use these strategies were female, minority and had lower education levels. Thus, demographic and socioeconomic disparities exist in the utilization of these strategies among Medicare Supplement insureds, likely due to lack of knowledge, expertise and/or communication with physicians.^14^

Among the cost-saving strategies utilized by older adults to reduce OOP spending, obtaining free samples from physicians was the most frequently used strategy in our study and in others.^17^^–^ 21 Levels of utilization of free samples, however, were generally about 50 % in representative overall Medicare populations, somewhat higher than the 39.6 % we demonstrated among this Medicare Supplement study group.^18^^,^ 19 ^,^ 21 This may be due in part to the higher socioeconomic status of Medigap beneficiaries (those with upper middle incomes were less likely to use cost-saving strategies).

Similarly, characteristics associated with the use of any cost-saving strategies among Medicare Supplement insureds were reflective of other overall Medicare studies.^18^^–^ 21 Those more likely to use the strategies were white, had more education, had more chronic conditions and disabilities and were taking more medications. These results support the findings that, while higher OOP spending levels encourage use of cost-saving strategies, those who did utilize the strategies apparently had more cost-management skills, knowledge and expertise in accessing these additional medication sources. In contrast, minorities and lower education individuals were less likely to use cost-saving strategies, or, in another study, request free samples or have conversations about medication costs with their physicians.^14^

Few variables significantly predicted medication nonadherence in our analysis. However, the strongest predictors included using more medications, splitting pills to make medications last longer, obtaining free samples from physicians and being male. As in other studies, with the exception of health status, few demographic or socioeconomic variables significantly predicted nonadherence.^10^^,^ 16 ^,^ 26 ^–^ 28 ^,^ 30 In our results, men were more likely to use untrackable cost-saving strategies, and consequently, were also more likely to be nonadherent. Consistent with most studies, taking more medications was highly predictive of nonadherence: as the cost burden of purchasing more medications increased, nonadherence also increased.^23^^–^ 27 ^,^ 29

Medication adherence is a complex behavior with few characteristics, beyond cost-related issues, that remain consistent across populations. Since reasons for medication nonadherence vary by individual, designing effective interventions to minimize medication nonadherence must also be multi-faceted. From the patient perspective, education efforts should focus on increasing disease-related knowledge and encouraging communications with physicians on cost-related issues.^15^^,^ 16 Reminder programs remain essential, since forgetting to take or refill medications are primary reasons for many older adults’ nonadherence.^30^^,^ 33 Prescription medication programs may need to incorporate culturally sensitive materials to address differences in knowledge, attitudes and health beliefs that influence medication decisions.^30^ From the provider perspective, greater awareness of patient prescription cost issues must inform prescription medication choices.^12^^,^ 15 ^,^ 33 In addition, physician, pharmacist or nurse drug review programs would be helpful in assessing overall drug protocols and/or the review of purchasing patterns for prescription medications.

### Limitations

Our study sample of AARP Medicare Supplement insureds included a sampling strategy to oversample those with health issues; thus, our results may not generalize to all Medicare Supplement populations. However, propensity weighting was utilized to adjust for non-response bias, so results should be generalizable to the sampled population. Also, the survey questions on cost-saving strategies did not query reasons for selected actions. For example, many free samples may have been used to try new medications rather than to address cost-related issues.^21^ Similarly, pill splitting can be a cost-saving strategy by design, with scored pills meant to be split, maintaining prescribed dosages, rather than self-determined splitting of pills to make medications last longer. Overall, although 92 % of survey respondents self-reported taking prescription medications, only 73 % had documented pharmacy claims in the Part D administrative database. This discrepancy is in line with expectations from the scientific literature,^7^ and may be due to either an overestimation of self-reported medication consumption,^7^^,^ 38 or an underestimation from pharmacy claims because of untrackable sources, under-reporting by retail outlets, or other insurance coverage of those medications.^7^

## CONCLUSION

Cost-saving strategies are used extensively by Medicare Supplement insureds to augment their Part D coverage. Disparities are evident in that females, minorities and those with lower education levels are less likely to benefit from cost-saving strategies. While use of alternative sources of prescription drugs can be helpful in managing OOP drug costs, these untrackable sources of prescription medications are associated with measured medication nonadherence. Pharmacy and case management programs should consider these additional medication sources in interventions that assist plan members in managing their medication protocols and in helping them to solve cost-related issues.
